# Population dynamics of microbial cross-feeding are determined by co-localization probabilities and cooperation-independent cheater growth

**DOI:** 10.1038/s41396-021-00986-y

**Published:** 2021-05-05

**Authors:** Rinke J. van Tatenhove-Pel, Daan H. de Groot, Anjani S. Bisseswar, Bas Teusink, Herwig Bachmann

**Affiliations:** 1 Systems Biology Lab, Amsterdam Institute of Molecular and Life Sciences, VU University Amsterdam, de Boelelaan 1108, Amsterdam, The Netherlands; 2 Department of Biotechnology, Delft University of Technology, Van der Maasweg 9, Delft, The Netherlands; 3 NIZO Food Research, Kernhemseweg 2, Ede, The Netherlands

**Keywords:** Community ecology, Microbial ecology, Evolution, Microbial ecology

## Abstract

As natural selection acts on individual organisms the evolution of costly cooperation between microorganisms is an intriguing phenomenon. Introduction of spatial structure to privatize exchanged molecules can explain the evolution of cooperation. However, in many natural systems cells can also grow to low cell concentrations in the absence of these exchanged molecules, thus showing “cooperation-independent background growth”. We here serially propagated a synthetic cross-feeding consortium of lactococci in the droplets of a water-in-oil emulsion, essentially mimicking group selection with varying founder population sizes. The results show that when the growth of cheaters completely depends on cooperators, cooperators outcompete cheaters. However, cheaters outcompete cooperators when they can independently grow to only ten percent of the consortium carrying capacity. This result is the consequence of a probabilistic effect, as low founder population sizes in droplets decrease the frequency of cooperator co-localization. Cooperator-enrichment can be recovered by increasing the founder population size in droplets to intermediate values. Together with mathematical modelling our results suggest that co-localization probabilities in a spatially structured environment leave a small window of opportunity for the evolution of cooperation between organisms that do not benefit from their cooperative trait when in isolation or form multispecies aggregates.

## Introduction

Cooperation is observed in many domains of life, from human social interactions [1] and cooperation in multicellular organisms [2], to cooperation between single-cell bacteria in ecosystems [3]. In the presence of cooperation, cheaters regularly emerge, showing “selfish” behaviour by taking benefit from the public goods produced by other community members, without paying the costs [1, 3–5]. The abundance of costly cooperation in natural systems is puzzling, as natural selection acts on individuals and is therefore expected to favour the selfish behaviour of cheaters.

Theories explaining the abundance of costly cooperation (from now on referred to as cooperation) often rely on the principle that benefits should preferentially be received by cooperators via group- or kin-selection, as in this way cooperators have a fitness advantage over cheaters [3, 6–9]. Group selection in microbial systems can be obtained by generating a spatially structured environment in which many sub-communities are formed, for example in a biofilm [3, 5, 8, 10–14]. Experimental validations of this theory show for example that growth on agar plates and compartmentalization in 96-well plates selects for cooperators, while growth in well-mixed suspensions selects for cheaters [10, 12, 13, 15]. Similarly, studies that compartmentalize and analyse many sub-communities in parallel can elucidate interactions in existing consortia [16, 17]. Growth in spatially structured environments can however also inhibit cooperation, for example when it increases competition between cooperators [8, 18–20].

Bidirectional cross-feeding is a specific type of cooperation, in which two cell-types mutually depend on each other [3]. In some cases, cooperator-pairs are obliged cross-feeders and growth of cheaters is completely cooperator dependent [10, 13]. In other cases, cheaters grow to low cell-concentrations in the absence of cooperators. For example, in the two species consortium used to produce yoghurt, species grow individually to a certain background concentration, while co-cultures are bidirectionally cross-feeding and reach higher final cell concentrations [21]. In other bidirectional cross-feeding communities (e.g., kefir, sourdough), this cooperation-independent “background growth” of monocultures is also reported [22, 23]. As natural ecosystems consist of many nutrients and many species [5], background growth is expected to be common.

Few studies analysed if and how this cooperation-independent background growth affects the outcome of competition between cheaters and mutually dependent cooperator-pairs. Müller et al. showed that in the presence of background growth expansion of spatially structured populations resulted in the loss of interaction between cooperators, but they did not include cooperator-cheater competition [24]. Evolutionary game-theory models sometimes include so-called “loners”, strains which do not affect the cooperation but are able to grow to a low cell concentration [25, 26]. However, as these loners are not involved in the cooperation, this is different from the background growth in for example the yoghurt consortium.

We here developed a high-throughput set-up that allows for cultivation of millions of subpopulation in parallel. Using this setup, we studied how cooperation-independent background growth affects the fitness of a bidirectional cross-feeding cooperator-pair and cheaters. We computationally predicted and experimentally measured the fractions of the different cell-types during repeated cycles of compartmentalized growth, with intermittent mixing of populations. This approach is similar to the haystack model [27–29], but we include non-clonal founder populations as starting points. Both the model and the experiments show that cooperators outcompete cheaters when cheater growth completely depends on cooperators. However, when cheaters grow to low cell concentrations in the absence of cooperators, they outcompete cooperators. We show that this is a consequence of how cooperator-pairs and cheaters are distributed over compartments in a spatially structured environment.

## Materials and methods

### Strains and media

All used strains are listed in Table 1. *L. lactis* was grown in chemically defined medium (CDM) described by Otto et al. [30], with the following changes: 7.5 g/L K_2_HPO_4_, 9 g/L KH_2_PO_4_, 0.6 g/L NH_4_-citrate, 2.5 mg/L biotin, 0.02 mg/L riboflavin and no folic acid (CDM_aa_). For growth in the presence of casein 5 g/L casein sodium salt (from bovine milk, C8654, Sigma-Aldrich, Saint Louis, MO, USA) was added to the medium instead of amino acids (CDM_cas_). For growth in the presence of casein and a limiting amount of amino acids (CDM_cas,aa_), 0.6% of the amino acid concentrations in CDM_aa_ were added to CDM_cas_. *L. lactis* NZ9000 Glc^-^Lac^+^ was pre-cultured in CDM_aa_ + 1.0 wt% lactose, *L. lactis* NZ5500 in CDM_aa_ + 0.5 wt% lactose and *L. lactis* MG610 in CDM_cas_ + 0.5 wt% glucose.

**Table 1:** Bacterial strains used in this study.

*L. lactis* strain	Description	Reference
NZ5500	*L. lactis* MG5267 with a loxP-ery-usp45p-melA-loxP fragment integrated into the genome. Erythromycin resistant.	[31]
NZ9000 Glc^-^Lac^+^	NZ9000*∆glk∆ptnABCD* containing a 657-bp deletion in *ptcBA*, carrying pMG8020 (lactose mini-plasmid of 23.7 kb, containing *lacFEGABCD*, derivative of pLP712). This strain grows on the galactose moiety but not on the glucose-moiety of lactose.	[32]
MG610	*L. lactis* MG1363 with two *prtMP* copies stably integrated into the genome. Erythromycin resistant.	[33]

### Propagation in suspension

Pre-cultures were washed and the cell-concentration in a SYBR^TM^ Green I (Thermo Fisher Scientific, Waltham, MA, USA) stained sample was determined using flow cytometry (Accuri C6, BD Biosciences, San Jose, CA, USA). Washed pre-cultures were diluted in CDM_cas_ + 0.5 wt% lactose or CDM_cas,aa_ + 0.5 wt% lactose to 3·10^6^ cells per mL. This concentration mimics the initial concentration in inoculated droplets in water-in-oil emulsions. Suspensions were incubated for 20 h at 30 °C while rotating to ensure well-mixed conditions. After growth the suspension was used for further analysis and propagation.

### Propagation in water-in-oil emulsion

Water-in-oil emulsions were made by mixing an oil phase and a water phase. The oil-phase contained Novec HFE 7500 fluorinated oil (3 M, Maplewood, MN, USA) and 0.2% PicoSurf1 surfactant (Sphere Fluidics, Cambridge, UK). The water-phase contained CDM_cas_ and cells, and it was prepared as follows. Pre-cultures were washed, and diluted in CDM_cas_ + 0.5 wt% lactose or CDM_cas,aa_ + 0.5 wt% lactose to 4.5·10^5^ cells per mL for a λ-value of 0.15 and to 6.0 ∙ 10^6^ cells per mL for a λ-value of 2. 300 µL water-phase and 700 µL oil-phase were added to a 10 mL tube and vortexed for 15 min in a tube rack on a vortex mixer at 2500 rpm. Most formed water droplets had a diameter of 90 µm (Fig. S1, supplementary information section 1). Emulsions were incubated for 20 h at 30 °C, without shaking. After incubation 300 µL CDM_cas_ + 0.5 wt% lactose or CDM_cas,aa_ + 0.5 wt% lactose and 1 mL perfluorooctanol (PFO, Alfa Aesar, Ward Hill, MA, USA) were added to the emulsion, which leads to the breaking of the emulsion upon gently mixing. The water phase, containing the cells, was separated from the oil phase and used for further analysis and propagation.

### Growth assays

The most probable number (MPN) procedure was used to determine concentrations of individual cell-types in the mixed cultures. In total, 8 replicates of a tenfold dilution series were scored for growth after at least 3 days of incubation in either CDM_aa_ with lactose and 10 µg/mL erythromycin (growth of *L. lactis* NZ5500 only), CDM_cas_ with glucose (growth of *L. lactis* MG610 only) or CDM_aa_ with lactose (growth of *L. lactis* NZ9000 Glc^-^Lac^+^ and *L. lactis* NZ5500). See Fig. S2 and supplementary information section 2 for a more detailed explanation. MPN counts were determined as described earlier [31].

### Probabilistic model

We modeled serial propagation of a consortium in water-in-oil emulsions. The model was implemented in Python. The maximal carrying capacity of compartments was set to 750 cells, based on a compartment-volume of 382 pL (supplementary information section 1) and a maximal final cell concentration in the used medium of 2·10^9^ cells/mL (Table S1).

The model consists of 5 steps, which are described in more detail in Fig. S3 and section 4 of the supplementary information. In short, we calculated the number of compartments inoculated with 0, 1, …, n cells according to a Poisson distribution. We subsequently determined all possible cell-type combinations and their probabilities, based on the initial cell-type fractions. Hereafter, we simulated growth in the compartments, for each possible cell-type combination. We subsequently calculated the probability that a cell of a certain cell-type ends up in a specific cell-type combination, and multiplied that probability with the growth factor that it would attain in that combination. By doing this for all cell-type combinations, we obtained an average growth factor for all cell-types. Finally, we used these growth factors to calculate the new cell-type fractions.

All computational results were obtained using Python, code is freely available on GitHub at https://github.com/dhdegroot/enrichment-of-cross-feeding-in-spatial-structure.

## Results

### When cheaters are cooperation-dependent, cooperator-enrichment is optimal at small founder populations sizes

**Fig. 1:**
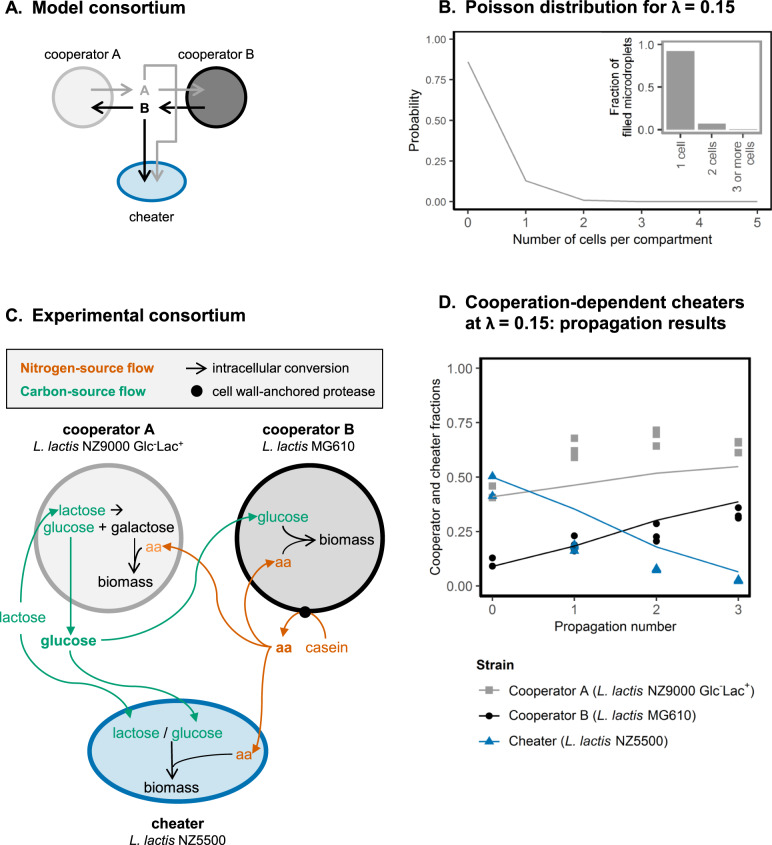
The modeled consortium. **A** Grey arrows indicate the flow of compound A, produced by cooperator A. Black arrows indicate the flow of compound B, produced by cooperator B. **B** Poisson distribution for a λ-value of 0.15. **C** The three *L. lactis* strains that formed the synthetic consortium. (1) “Cooperator A” uses lactose as carbon source and peptides as nitrogen source. It secretes glucose. (2) “Cooperator B” uses glucose as carbon source. It extracellularly degrades casein into peptides, which it can use as nitrogen source. (3) “Cheaters” use lactose and glucose as carbon source, and peptides as nitrogen source. **D** Propagation results. The synthetic consortium shown in (**C**) was propageted in water-in-oil emulsion, at a λ-value of 0.15. **D** shows the experimental results (points, *n* ≥ 2) and corresponding model predictions (lines). Note that all model parameters were measured experimentally, so that none of them was used to fit the model’s predictions to the experimental outcomes. From propagation 0 to propagation 3 the fractions of cooperator A and cooperator B were significantly increased (*p* < 0.001 and *p* = 2.0 ∙ 10^-2^ respectively), and the fraction of cheaters was significantly decreased (*p* < 0.001).

We made a probabilistic model to study competition between cooperators and cheaters. This model simulated repeated cycles of compartmentalized growth with intermittent mixing of populations. The consortium consists of a bidirectional cross-feeding cooperator-pair (cooperator A and cooperator B), and a non-producing cheater that consumes the cross-fed compounds (Fig. 1A).

Using this probabilistic model, we first analysed cooperator and cheater interactions when cheaters need the public goods produced by the cooperators to grow—cooperation-dependent cheaters. The λ-value of the Poisson distribution is equal to the average number of cells per compartment, and it represents therefore the average founder population size. We computationally varied the λ-value and the initial cooperator and cheater fractions, to predict which average founder population size results in rapid cooperator enrichment. We found optimal λ-values between 0.15 and 0.76 (Fig. S4). Higher initial cheater fractions resulted in higher optimal λ-values, as increasing the average founder population size ensures that compartments contain cooperator-pairs. Yet when the initial cooperator fraction is high, reducing the founder-population size (lower λ) ensures that the cheaters do not consume the public goods produced by cooperators. Because the cooperator fraction increases after every propagation, the optimal λ is expected to decrease after every propagation. We decided to use the lowest optimal λ (*λ* = 0.15) in subsequent modelling studies and experiments; Using this λ-value maximizes the number of compartments with two cells while minimizing the number of compartments with three or more cells (95% of the compartments with two or more cells contains two cells, Fig. 1B). At this small founder populations size cooperators are expected to outcompete cooperation-dependent cheaters.

### Serial propagation of a synthetic consortium confirms the predicted cooperator-enrichment

We experimentally validated these model predictions by constructing a synthetic bidirectional cross-feeding consortium based on three *L. lactis* strains (Fig. 1C). “Cooperator A” (*L. lactis* NZ9000 Glc^-^Lac^+^) takes up lactose as carbon source and hydrolyses it intracellularly to glucose and galactose. It was engineered to not metabolize glucose, so it secretes glucose while it metabolizes galactose [32]. It uses peptides as nitrogen source. “Cooperator B” (*L. lactis* MG610) takes up glucose as carbon source. It can use casein as its nitrogen source, as it expresses an extracellular protease which degrades casein into peptides [33]. The “cheater” (*L. lactis* NZ5500) uses both lactose and glucose as its carbon source, and peptides as its nitrogen source [34].

In a medium with lactose and casein as the respective carbon and nitrogen source, cooperator A and B are obligatory cross-feeders, and cheaters only grow in the presence of cooperators. In these conditions the system therefore represents a consortium with cooperation-dependent cheaters.

We propagated a mixture of cooperator A, cooperator B and cheaters in water-in-oil emulsions to simulate repeated cycles of compartmentalized growth with intermittent mixing of populations. Consistent with the model predictions, we observed cooperator enrichment in these conditions (Fig. 1D). Propagation of the same consortium in a non-structured suspension (founder population size of ~10^7^ cells), resulted in cheaters outcompeting cooperators and the population growth was reduced (Fig. S5). This difference in cooperator and cheater fitness is most likely caused by the difference in the λ-value (founder population size), as *L. lactis* is not very sensitive to variations in oxygen and stirring itself is not known to alter its physiology [35, 36]. Together these results confirm that cooperators are enriched when cheaters are cooperation-dependent and when founder population sizes are small.

### When the founder population size is small, cooperation-independent background growth hampers selection for costly cooperation

**Fig. 2:**
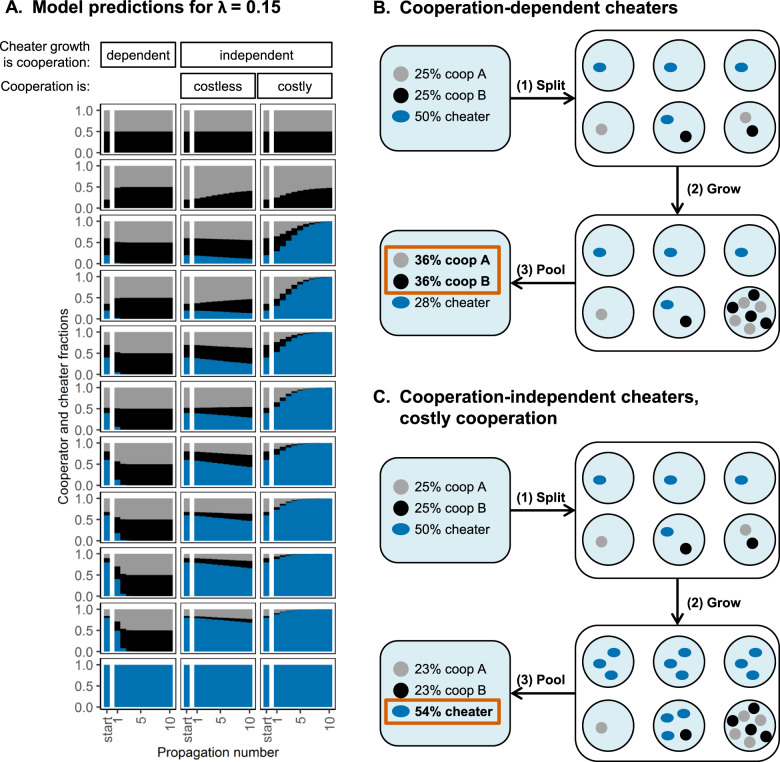
Enrichment predictions for a λ-value of 0.15. **A** We simulated growth during serial propagation in a spatially structured environment, with a λ-value of 0.15. **A** shows the cooperator and cheater fractions (y-axis) in time during these propagations (x-axis). Each row corresponds to a different set of initial cooperator and cheater fractions, indicated by “start” on the x-axis. Each column corresponds to a different simulation scenario. First column: cheaters need public goods produced by cooperators to grow (dependent). Second column: cooperation-independent background growth of all cell-types up to 20% of the maximal carrying capacity (independent, costless). Third column: cooperation-independent background growth of all cell-types, but individual cooperators reach 10% of the maximal carryinig capacity while individual cheaters reach 20% (independent, costly). **B**, **C** Schematic overview of cooperator and cheater growth in a spatially structured environment with a low average founder population size. Starting point is a population with 50% cheaters. Cells are randomly distributed over compartments (1), allowed to grow (2) and subsequently pooled (3). In (**B**) cheaters are cooperation-dependent. We here assumed that cheaters need the public goods produced by cooperators to grow. In this scenario cooperators outcompete cheaters. In (**C**) cheaters grow independently of cooperators to low cell concentrations. We here assumed that cheaters could reach low cell concentrations in the absence of cooperators. In this scenario cheaters outcompete cooperators.

After showing that cooperators are enriched when cheaters are cooperation-dependent and founder population sizes are small (Fig. 1, Fig. 2A first column, Fig. 2B), we modeled two more scenarios. In these scenarios we analyse how cooperation-independent background growth affects competition between cooperators and cheaters during repeated cycles of compartmentalized growth with intermittent mixing of populations.

In the first scenario, all cell-types could independently reach 20% of the maximal carrying capacity of the compartment, while the full carrying capacity was only reached in the presence of a cooperator-pair. Under these conditions the model predicted enrichment of the cooperators for all tested initial cooperator and cheater fractions, although this enrichment was slow compared to conditions in which all cell-types completely depended on public goods produced by the cooperators to grow (Fig. 2A, second column).

In the second scenario all cell-types could still independently reach a low cell concentration, but we assumed that cooperation was costly and individual cooperators therefore reached only 10% of the maximal carrying capacity of the compartment, while cheaters still reached 20% (Supplementary information section 4, Fig. S6). Under these conditions, the model predicted that cheaters outcompeted cooperators for all tested initial cooperator and cheater fractions (Fig. 2A, third column). This loss of cooperation is caused by the random nature of distributing the cells over different compartments combined with a low founder population size, which results in a higher fraction of compartments containing cheaters than containing a cooperator-pair. So, even though cooperator cells have a large advantage in some compartments, cheater cells have a small advantage in many compartments, allowing cheaters to outcompete cooperators.

Altogether these results indicate that when cheaters need the public goods produced by cooperators to grow, costly cooperation can be enriched when the founder population size is small. However, this selective advantage completely vanishes when cheaters can reach low cell concentrations in the absence of cooperator-pairs. Figure 2C schematically summarizes these findings.

### Cross-feeding cooperator-pairs outcompete cheaters in spatially structured environments with an intermediate average founder population size

**Fig. 3:**
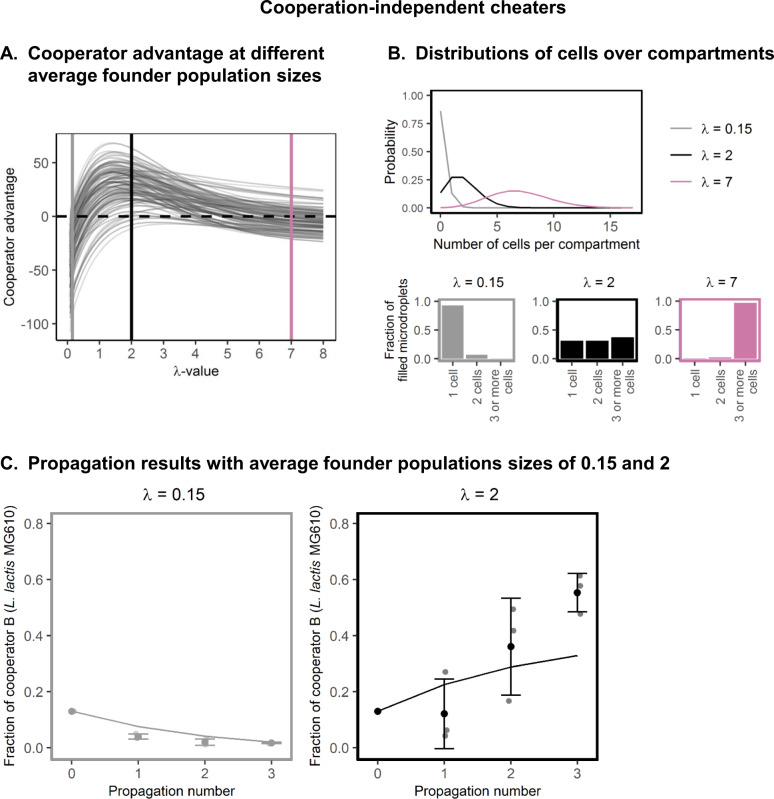
The effect of the average founder population size on the cooperator advantage, in the presence of cooperation-independent background growth. **A** Shows the λ-value versus the cooperator advantage (given by the difference in growth factor of the cooperator and cheaters, see supplementary information section 4). The dashed horizontal line indicates a cooperator advantage of 0. Different grey lines correspond to a different randomly-sampled parameter set. Coloured vertical lines indicate a λ-value of 0.15, 2 and 7. All model parameters were randomly chosen: initial cell-type fractions, cheater background growth (uniform from 20 to 120 cells), cooperator background growth (uniform from 0 to the cheater background growth), growth advantage of the cheater (normally distributed around s_c_ = 0.40 with standard deviation of 8%). **B** Poisson distributions for different λ-values. Poisson distribution for *λ* = 0.15, *λ* = 2 and *λ* = 7 corresponding to the coloured lines in (**A**) are shown. The line-plot shows the probability distributions for all three λ-values, the bar-plots show which fraction of filled compartments contains 1, 2 and 3 or more cells. **C** Propagation results. The synthetic consortium shown in Fig. 1A was propageted in water-in-oil emulsion, at a λ-value of 0.15 or 2. A growth-limiting amount of amino acids was present in the medium, to allow cooperation-independent background growth of cheaters. This panels shows the experimental results (mean ± sd, *n* = 3, opaque points show the individual measurements) and corresponding model predictions (lines). From propagation 0 to propagation 3 the fraction of cooperator B was significantly decreased for *λ* = 0.15 (*p* < 0.001), and significantly increased for *λ* = 2 (*p* = 8.8 ∙ 10^-3^).

We varied the λ-value of the Poisson-distribution and predicted which average founder population size leads to cooperator enrichment in the presence of cooperation-independent background growth. This simulation was performed 4000 times with randomly sampled parameters (supplementary information section 4), to ensure that the obtained result was valid for different cooperation costs and benefits. In these simulations the background growth of cooperator A and cooperator B were independent from each other, and varied from 0 to the same level as the cheater background growth. Figure 3A shows the cooperator advantage as a function of the average founder population size (the λ-value). We identify three different regimes. If the average founder population is too small (λ < 1), the cooperator cells cannot improve the cooperator-fitness in their compartment significantly, because they are rarely co-localized with their cooperation partner (Fig. 3B). If the average founder population size is too large (λ ≈ 10), almost all compartments will have many co-localized cooperator-pairs, and the individual contributions do not significantly improve the compartment’s growth conditions (Fig. 3B). When on average one or two cells interact with each other (λ-value between one and two), the probability for a cheater cell to be co-localized with a cooperator-pair is low: the cheater should be in a compartment with minimally three cells and at least one cooperator-pair, which is rare at a λ-value of 2. At the same λ-value the probability for a cooperator to be part of a cooperator-pair is much larger: the cooperator should be in a compartment with minimally two cells from which at least one is its opposite cooperator-type, which is more common at a λ-value of 2. Therefore, even though cheaters produce more offspring than cooperators in each compartment where they occur, overall the cooperators will outcompete the cheaters. This selective advantage that cooperators gain through encounter statistics was first described in the seventies [37], and is nowadays often referred to as the ‘Simpson’s paradox’ [15].

To experimentally verify these results, we added a growth-limiting amount of amino acids to CDM_cas_ (CDM_cas,aa_). Under these conditions there is cooperation-independent nitrogen-limited background growth of cooperator A and the cheater, up to 10% of the maximal carrying capacity of the compartment (supplementary information section 3). We propagated a cooperators-cheater mixture in this medium, using different average founder populations sizes. As cooperator A and the cheater reach similar cell concentrations in the absence of cooperator-pairs we cannot distinguish between them in our most probably number assay (supplementary information section 2). We therefore used the fraction of cooperator B as read-out for cooperator enrichment. When the average founder population size was small (*λ* = 0.15), cooperator B was outcompeted, consistent with the model predictions (Fig. 3C). However, at an intermediate founder populations size (*λ* = 2) cooperator B increased in frequency, consistent with the model predictions (Fig. 3C). In non-structured environments the founder population size (“λ-value”) was far above 2, and the cheater outcompeted cooperators (Fig. S5).

### The effect of cooperation costs and benefits on cooperator-fitness

**Fig. 4:**
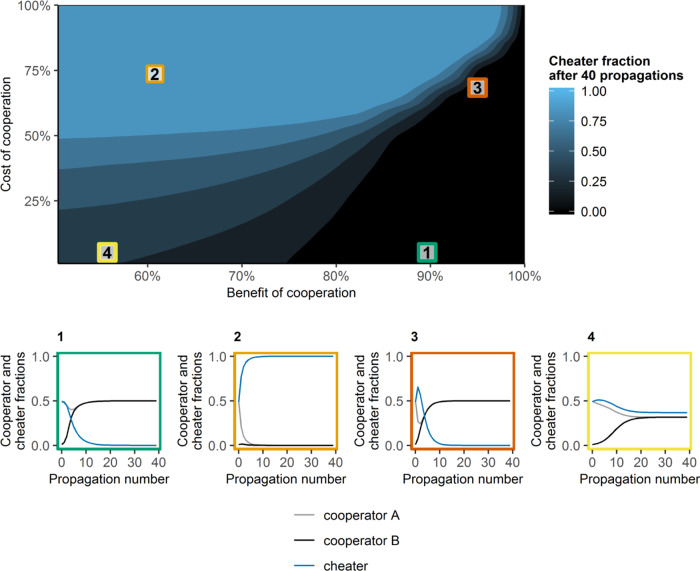
Cheater fractions after 40 propagations at λ = 2. Each simulation started with 50% cooperator A, 1% cooperator B, and 49% cheater. Simulations were repeated 40 times to model serial propagation. On the x-axis, we vary the advantage of cooperation, which is given by the fraction of the maximal carrying capacity of the compartment that can only be filled in the presence of cooperator-pairs. On the y-axis, we vary the cost of cooperation, which is given by the percentage difference in independent growth of cooperators and independent growth of cheaters. The colour gradient quantifies the predicted cheater fraction after 40 propagations. For four different regimes in this plot, we show how the cell-type fractions change with the number of propagations.

Even when the optimal average founder population size is used, cooperator-pairs do not always outcompete cheaters. We did simulations where we started with 50% cooperator A, 1% cooperator B, and 49% cheater. The cheater fraction after 40 propagations was predicted for (i) different choices of cooperation benefits (carrying capacity of the compartment in the presence of a cooperator-pair), or (ii) different costs of cooperation (difference between independent growth of cheaters and independent growth of cooperators, where cooperator A and cooperator B had the same level of independent growth). We found that when the benefit of cooperation is high and the cost of cooperation is low (regime 1), cooperators are enriched (Fig. 4). In this region, especially when the cost of cooperation is 0, enrichment might be faster with a lower average founder population size, as shown in Fig. 1. When the cost of cooperation is high and the benefit of cooperation is low (regime 2), cheaters are enriched. When both the cooperation benefits and costs are high (regime 3), there is a sudden switch between cheater or cooperators enrichment, depending on the actual costs and benefits. When the benefits and costs of cooperation are both low (regime 4), cheaters and cooperators coexist. This is because two effects keep each other in balance. First, at a high cheater fraction, cheaters are less likely to be co-localized with cooperator-pairs and, as cooperation-costs are low, cooperators are enriched. However, when the initial fraction of cheaters decreases, they will increasingly co-localize with cooperator-pairs, and, as cooperation-benefits are low, the cheater cells will be enriched. Serial propagation of this system will therefore lead to a stable co-existence of cooperators and cheaters.

In regimes 3 and 4 the ratios between the costs and benefits of cooperation are similar (high cost/high benefit in regime 3, low cost/low benefit in regime 4), but the predicted population dynamics are different. This difference might be explained by the difference in background growth between regime 3 and 4: When the background growth is low compared to the growth in compartments with cooperator-pairs (regime 3), a small change in costs or benefits of cooperation have a stronger effect on the outcome of the competition than when the background growth is high compared to growth in compartments with a cooperator-pair (regime 4).

Together, these results show that even at the optimal founder population size, cooperators can only outcompete cheaters when the cost/benefit ratio of cooperation is large enough.

## Discussion

We show that during serial propagation in a spatially structured environment the window of opportunity for the selection of mutualistic cross-feeding interactions is small: when the average founder population size is too high or too low, cheaters outcompete cooperators. This is caused by a probabilistic effect as during the random distribution of individual cells over a spatially structure environment, for example during biofilm dispersal [38], the chance that cooperators encounter each other are reduced. Our results subsequently point towards several cooperation-stabilizing factors that might increase the fitness and stability of mutualistic interactions in natural consortia.

We show that cooperator enrichment requires cooperator cells to significantly improve the cooperator-fitness within their compartment. We used a bidirectionally cross-feeding synthetic consortium in which cooperators only benefit from their cooperative phenotype when they form a pair with another cooperator cell-type, a so-called “prisoner’s dilemma” interaction [5, 19]. In these conditions individually compartmentalized cooperators do not have a fitness advantage of their cooperative phenotype. However, a second type of cooperation is described by the so-called “snowdrift game” interaction, in which cooperators can direct part of the benefits of their public goods to themselves [19, 39]. This is for example the case for interactions involving extracellular enzymes, where both the enzyme producer and, when present, its neighbours benefit from the released substrate [4, 21, 40, 41]. A similar situation occurs when the cooperator is a single strain instead of a cooperator-pair. For example strains with a high biomass yield and low growth rate are sometimes referred to as single strain cooperators [12, 14, 42]. These systems are similar to the snowdrift game, as these cooperators only interact with their kin and colocalization of a cooperator-pair is not required. In such snowdrift game type of conditions, individually compartmentalized cooperators do have a fitness advantage of their cooperative trait. Our results therefore suggest that these “snowdrift game” type of interactions might form a more stable basis for the evolution of mutualistic interactions, as they are less affected by variation in the founder population size. Previous studies indicate that in such systems at low founder population sizes cooperators can outcompete cheaters [12, 40].

For “prisoner’s dilemma” type of interactions cooperators can increase their fitness by increasing their chance of co-localization, for example by forming aggregates. Costless cooperating organisms have for example been shown to evolve cell-aggregation [43, 44], and it is reported that mutualists can only successfully co-expand into new territories when the cooperation benefits are large, or when the cooperators are physically linked to each other [24].

Our experimental setup allowed for background growth of one of the cooperators and the cheater, and our results show a small window of opportunity for the selection of cooperation. The simulations show that this small window of opportunity holds for all combinations of cooperator and cheater background growth, as long as the background growth of at least one of the cooperators is lower than that of the cheaters. However, when the background growth of cooperators and cheaters is the same (i.e., cooperation is costless), culturing in a spatially structured environment allows for cooperator enrichment, even in the presence of cooperation-independent background growth of cheaters. This supports the previously raised hypothesis that costless secretion of metabolic products might be the starting point for evolution of costly cooperation [3, 8, 10, 43, 45–47].

Cells can also use more complex ways to increase the relative fitness of cooperators within the same compartment. Pillai et al. showed that in a consortium consisting of cooperators, weak cheaters and strong cheaters, cooperators could only persist when they stimulated the weak cheaters’ growth, allowing the weak cheaters to outcompete the strong cheaters [48]. Inglis et al. showed that cooperators can persist in a well-mixed system when cooperation-independent strains (so-called “loners”) outcompete cheaters that would otherwise outcompete cooperators [25]. Furthermore, cooperators can produce antimicrobials or toxins, to inhibit growth of cheaters in the same compartment [8]. These examples show that cooperators can increase the cooperator-fitness in their compartment in several ways, which leads to more complex interactions. This complexity is further increased when multiple cheater types are present per single cross-feeding reaction (e.g., one for public good A, one for public good B, one for both).

Cultivation in microdroplets is a laboratory environment, and growth of microbial consortia might not occur as controlled and structured in natural systems. However, the underlying principle of compartmentalized growth with intermittent mixing of populations is likely to occur in natural systems. Several recent studies highlight the small interaction-range of microorganisms [49, 50]. This results in compartmentalization of microbial growth and interactions even in the absence of a physical barrier. Furthermore, during biofilm dispersion single, planktonic cells are released to inoculate new environments [38], resulting in intermittent mixing of populations with small founder population sizes. In natural systems the volumes of these new environments are expected to differ from each other. In our experiments microdroplets were polydisperse (the droplet volume varied tenfold), partially capturing this variation.

In the past years several tools were developed to identify interactions in consortia, with the ultimate goal to better understand microbial communities [16, 17]. The drawback of most methods is that they require fluorescent protein expression to identify the different cell-types, a characteristic that is typically unavailable in natural communities. Serial propagation in water-in-oil emulsions can be used to select individual strains that reach high cell concentrations [27], and, as emulsions can contain millions of compartments per mL, to form many different community-compositions in parallel [16, 17, 51]. We here combined these properties and used serial propagation in emulsion to enrich bidirectionally cross-feeding cooperator-pairs from a synthetic consortium. As this approach does not require fluorescence labelling, it should allow for the elucidation of cooperative interactions in natural communities. In laboratory experiments, direct selection of compartments with the highest cell-concentration might further increase the selection efficiency [17, 52–54], as it eliminates the contribution of compartments with a low cell concentration.

Natural selection acts on the level of individual organisms, making evolution and stability of cooperative interactions an intriguing puzzle. We here developed an experimental setup that allows the cultivation of millions of sub-communities in parallel. Using this platform, we show that cooperation-independent background growth adds another layer of complexity to this puzzle, and that it results in founder population sizes affecting the fitness of cooperator-pairs and cheaters. Such background growth is expected to play an important role in natural environments such as soil or gastro intestinal tracts, where complex substrates are utilized by organisms with versatile metabolic capacities. On a different scale similar patterns are observed in populations of white-faced monkeys, free-ranging dogs and wolves, where the group-size determines whether cheating in collective actions such as hunting or territory defending is beneficial or not [55]. Cooperators can overcome these constraints by preventing the founder population size to become too high or too low [43, 55, 56], or, when the founder population size is low, by directing part of the benefits of their public good to themselves [39, 56].

## Supplementary information

Supplementary information
