# Supplementary material for: Population dynamics of microbial cross-feeding are determined by co-localization probabilities and cooperation-independent cheater growth
Source: ISME J. 2021 May 5;15(10):3050–61. doi: 10.1038/s41396-021-00986-y (PMC8443577; doi:10.1038/s41396-021-00986-y)
Supplement: Supplementary file 1 — Supplementary information [file 41396_2021_986_MOESM1_ESM.pdf]

Supplementary information for the manuscript entitled "Population dynamics of microbial cross-feeding are determined by co-localization probabilities and cooperation-independent cheater growth"

Rinke J. van Tatenhove-Pel[a,b], Daan H. de Groot[a], Anjani S. Bisseswar[a], Bas Teusink[a], Herwig Bachmann[a,c*]

[a] Systems Biology Lab, Amsterdam Institute of Molecular and Life Sciences, VU University Amsterdam, de Boelelaan 1108, 1081HV Amsterdam, The Netherlands

[b] Department of Biotechnology, Delft University of Technology, Van der Maasweg 9, 2629HZ, Delft, The Netherlands

[c] NIZO Food Research, Kernhemseweg 2, 6718ZB Ede, The Netherlands

* Address correspondence to Herwig Bachmann, h.bachmann@vu.nl.

Supplementary information

Section 1: Droplet size- and volume-distributions

We prepared water-in-oil emulsions and made pictures with a microscope (9 per emulsion, Figure S1A shows an example). Pictures were analysed with ImageJ to identify droplets (Figure S1B). Small droplets were not always identified, but as they contain only little volume this only marginally affects the analysis. Droplets on the edge of the picture were excluded from the analysis. Based on the ImageJ results the droplet size distribution was calculated (Figure S1C). When cells are homogenously spread in the water-phase, their distribution over droplets follows the droplet-volume distribution. We therefore calculated which droplet diameter contained the highest fraction of the total volume (Figure S1D), as most cells end-up in droplets with this diameter. Formed emulsions were polydisperse, but distributions of replicates were reproducible. The mean diameter ± SEM of droplets that contained the highest fraction of the total volume was 86±15 µm.

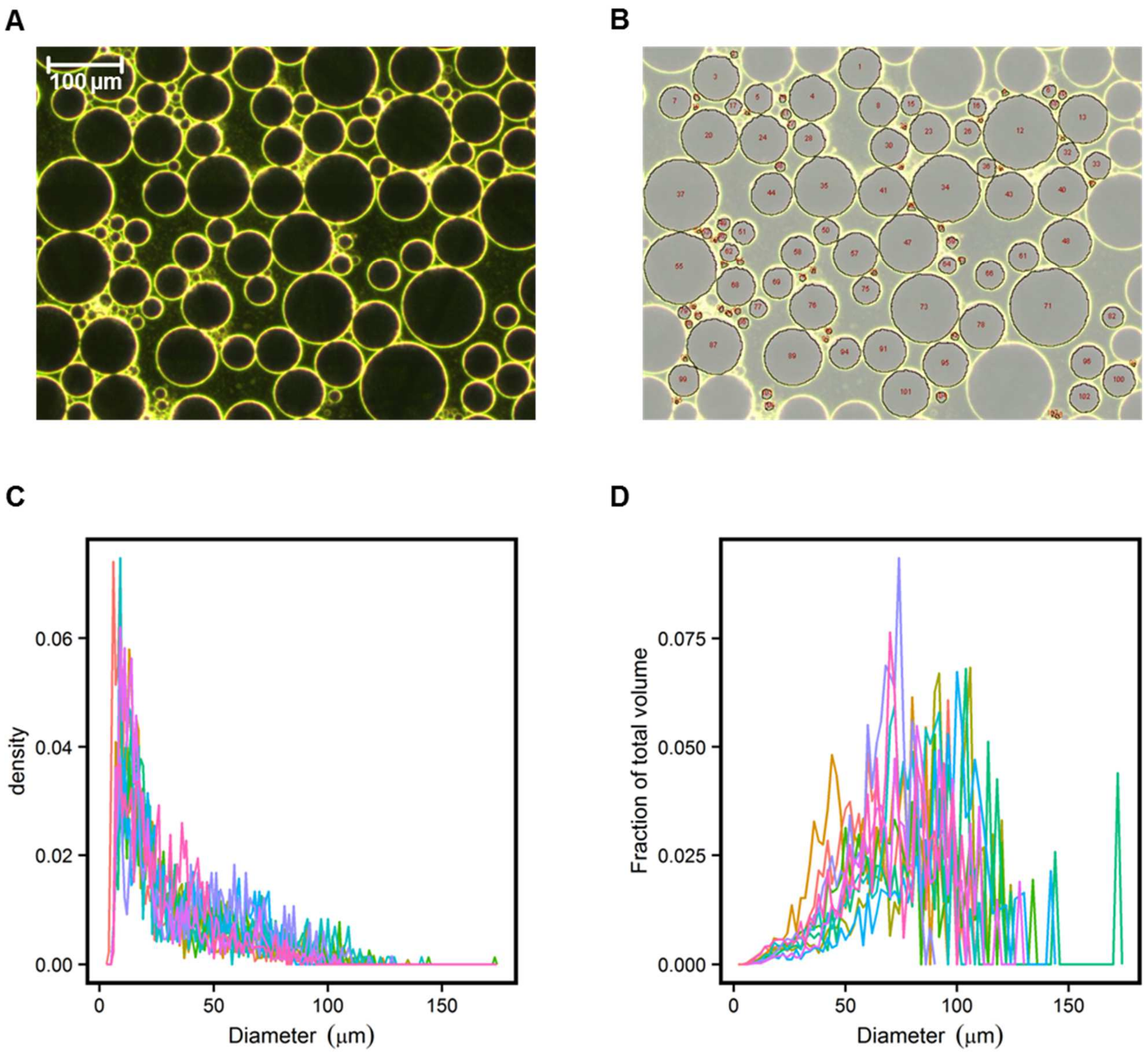

Figure S1. Droplet size and volume. (A) An example microscope picture of a water-in-oil emulsion. **(B)** Droplets identified in (A) after ImageJ analysis. **(C)** Droplet size distributions (n=10 emulsions, 9 pictures per emulsion). **(D)** The fraction of the total volume that is occupied by a given droplet diameter. The used bin width is 2 µm and the fraction was calculated for each distribution individually (n=10 emulsions, 9 pictures per emulsion).

Section 2: MPN calculations and raw MPN data

Figure S2A gives a schematic overview of the MPN measurements we did, and how they provide information on the cell concentrations of *L. lactis* MG610, *L. lactis* NZ9000 $Glc^{-}Lac^{+}$ and *L. lactis* NZ5500.

6.1. Calculating the fraction of *L. lactis* MG610

The cell concentration of *L. lactis* MG610 is given by the MPN in CDM_{cas} + glucose. The cell concentration of the two remaining strains, *L. lactis* NZ9000 $Glc^{-}Lac^{+}$ and *L. lactis* NZ5500, is given by the MPN in CDM_{aa} + lactose. Therefore the total cell concentration (all three strains together) is given by the MPN in CDM_{cas} + glucose plus the MPN in CDM_{aa} + lactose. This total cell concentration is used to calculate relative abundance of *L. lactis* MG610, by dividing the cell concentration of *L. lactis* MG610 by the total cell number.

6.2. Calculating the fractions of *L. lactis* NZ9000 $Glc^{-}Lac^{+}$ and *L. lactis* NZ5500

To obtain the individual fractions of *L. lactis* NZ9000 $Glc^{-}Lac^{+}$ and *L. lactis* NZ5500, we need to split their combined MPN in CDM_{aa} + lactose into the individual cell concentrations for both strains. The individual cell concentration of *L. lactis* NZ5500 is given by the MPN in CDM_{aa} + lactose + erythromycin. The individual cell concentrations of *L. lactis* NZ9000 $Glc^{-}Lac^{+}$ is subsequently calculated by subtracting the cell concentration of *L. lactis* NZ5500 from the cell concentration of *L. lactis* NZ9000 $Glc^{-}Lac^{+}$ and *L. lactis* NZ5500 together. When the concentration of *L. lactis* NZ9000 $Glc^{-}Lac^{+}$ and *L. lactis* NZ5500 are in the same order of magnitude, you would expect the MPN in CDM_{aa} + lactose to be twice as high as the MPN in CDM_{aa} + lactose + erythromycin. However, as Figure S2B shows this is within the measurements error of the MPN method, and therefore the two strains cannot be separated accurately. In Figure 3, we therefore only report the fractions of *L. lactis* MG610.

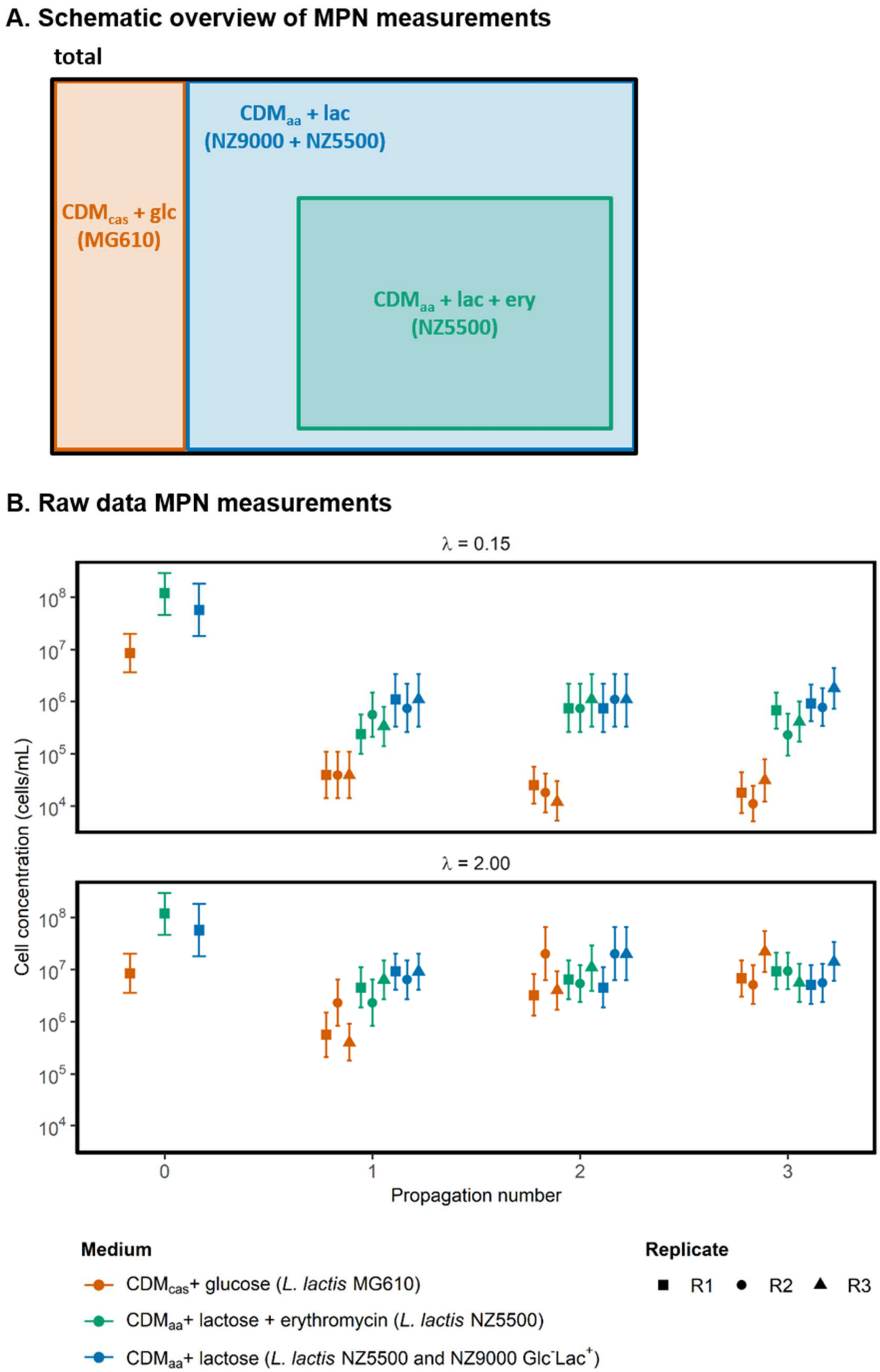

Figure S2. MPN calculations and raw MPN data. (A) Schematic overview of the selective media that was used for the MPN determinations. Strains that could grow on the specified medium are

indicated between brackets. **(B)** Raw data of the MPN measurements. For each propagation the 95% confidence limits (cells/mL) for each selective medium is shown.

Section 3: Mono- en co-culture analysis

L. lactis NZ9000 Glc$^-$Lac$^+$, *L. lactis* MG610 and *L. lactis* NZ5500 were cultured in CDM$_{cas}$ + 0.5 wt% lactose. The cell concentration in stationary-phase cultures was determined using flow cytometry (Table S1). Due to the presence of a small amount of free amino acids in casein, *L. lactis* NZ5500 and *L. lactis* NZ9000 Glc$^-$Lac$^+$ could independently grow to 1-2% of the maximal carrying capacity of the medium. In the model predictions for Figure 1D the cooperation-independent background growth of *L. lactis* NZ5500 and *L. lactis* NZ9000 Glc$^-$Lac$^+$ was therefore set to 1% of the maximal carrying capacity of the compartment.

In CDM$_{cas,aa}$ + 0.5 wt% lactose the amino acid concentration was set such that *L. lactis* NZ5500 reaches 10% of the final cell concentration of the *L. lactis* MG610 and *L. lactis* NZ9000 Glc$^-$Lac$^+$ co-culture (the cooperators). In the model predictions for Figure 3C the cooperation-independent background growth was therefore set to 10% of the maximal carrying capacity of the compartment.for *L. lactis* NZ5500 and to 7% for *L. lactis* NZ9000 Glc$^-$Lac$^+$ (Table S1).

Table S1. Stationary-phase cell concentrations. The stationary-phase cell concentration in CDM$_{cas}$ + 0.5 wt% lactose and in CDM$_{cas,aa}$ + 0.5 wt% lactose was measured for monocultures of cooperators and cheaters, and co-cultures of cooperators (n=3). Cultures were inoculated with approximately 10^6 cells/mL.

Strain	**CDM$_{cas}$**		**CDM$_{cas,aa}$**	
	Final cell concentration (cells/mL)	Percentage of maximal carrying capacity	Final cell concentration (cells/mL)	Percentage of maximal carrying capacity
L. lactis NZ9000 Glc$^-$Lac$^+$	$2.1 \cdot 10^7 \pm 0.1 \cdot 10^7$	1%	$1.1 \cdot 10^8 \pm 0.0 \cdot 10^8$	7%
L. lactis MG610	$2.5 \cdot 10^7 \pm 0.0 \cdot 10^7$	2%	$3.7 \cdot 10^7 \pm 0.1 \cdot 10^7$	2%
L. lactis NZ5500	$3.1 \cdot 10^7 \pm 0.0 \cdot 10^7$	2%	$1.5 \cdot 10^8 \pm 0.0 \cdot 10^8$	10%
L. lactis MG610 + *L. lactis* NZ9000 Glc$^-$Lac$^+$	$1.4 \cdot 10^9 \pm 0.2 \cdot 10^9$	100%	$1.6 \cdot 10^9 \pm 0.0 \cdot 10^9$	100%

Section 4: Probabilistic model

Figure S3 gives an overview of the probabilistic model that was implemented in Python. The model consists of 5 steps, which are described in section 4.1. The mathematical equations of these steps can also be found in section 4.2.

4.1. General description of the model

In Step 1, the expected number of droplets inoculated with 0, 1, …, n cells was calculated, assuming that the cells were randomly distributed over droplets with equal volumes. The expected number of droplets with k cells is then given by a Poisson distribution with parameter λ equal to the number of cells divided by the number of droplets.

In Step 2, given a number of cells per droplet, say k, all possible cell-type combinations were determined. This comes down to enumerating all ordered triplets of nonnegative integers that sum op to k. The probability of occurrence of each combination was calculated using a multinomial distribution with probabilities given by the initial fractions of the different cell-types. Using this probability and the number of droplets inoculated with 0, 1, …, n cells, the expected number of droplets with a specific cell-type-combination was calculated.

In Step 3, we simulated growth within each droplet. Cell death was not included in the model, so that the minimal growth factor was set to 1. The extent to which the different cell-types grew depended on the cell-types that were present in the droplet, and differed per incubation condition as explained in the main text. After simulating growth, all droplets were pooled and the total cell-number for each cell-type was calculated.

In Step 4, we calculated the expected growth factor of each cell-type. We first calculated for each cell-type the probability that it ended up in a specific cell-type combination. By multiplying this probability with the growth factor that it would attain in this combination, and then summing over all cell-type combinations, we obtained the expected growth factor of

this cell-type. Growth factors were determined by linearly interpolating between two extreme cell-type combinations: 1) no cooperator-pair was present, 2) the compartment almost exclusively contained cooperator-pairs. In the first case, the growth factor for each cell-type was determined completely by its cooperator-independent background growth. In the second case, the eventual total number of cells was set to the maximal carrying capacity of the compartment, and the maximal increase of the cheater fraction was set to 10% ($s_c = 0.40$), which is close to the experimentally measured value (supplementary information section 6). Furthermore, the different cooperator types were assumed to end up in a 1:1-ratio, reflecting the biological observation that they are stoichiometrically coupled by their cross-feeding behaviour (Figure 1A and description in main text).

In Step 5, we use these growth factors to calculate the new cell-type fractions. In the case that we simulated several propagations, these new cell-type fractions were used as an input for the next simulation.

4.2. Mathematical details

We describe a microbial community of three cell-types: cooperator A, cooperator B, and cheater C. The fractions of these cell-types in the initial population are denoted by f_a, f_b, f_c, respectively. The cells in the population are distributed over droplets (micro-environment), where the number of cells in one droplet is Poisson-distributed with mean λ:

$$P(N_t = n_t) = \frac{\lambda^{n_t} e^{-\lambda}}{n_t!}$$

The cell-types are distributed over droplets according to a multinomial distribution. So, given that a certain droplet has n_t cells, we have

$$P(N_a = n_a, N_b = n_b, N_c = n_t - n_a - n_b \mid N_t = n_t) = \binom{n_t}{n_a, n_b, n_t - n_a - n_b} f_a^{n_a} f_b^{n_b} f_c^{n_t - n_a - n_b}.$$

Therefore, the probability of finding a certain droplet is given by

$P(N_a = n_a, N_b = n_b, N_c = n_t - n_a - n_b)$

$= P(N_t = n_t)P(N_a = n_a, N_b = n_b, N_c = n_t - n_a - n_b \mid N_t = n_t)$

$= \frac{\lambda^{n_t} e^{-\lambda}}{n_t!} \binom{n_t}{n_a, n_b, n_t - n_a - n_b} f_a^{n_a} f_b^{n_b} f_c^{n_t - n_a - n_b}$

Now if we pick one cell of type A, what is the probability that it is in a droplet of a certain composition? If the number of droplets is N_D, we know that the number of cells of type A is given by $N_D \lambda f_A$. Then, we only have to know how many cells of type A occur in a droplet of the given composition. This is just the number of droplets of that type times the number of cells of type A in that type of droplet. This is given by

$N_C n_a \frac{\lambda^{n_t} e^{-\lambda}}{n_t!} \binom{n_t}{n_a, n_b, n_t - n_a - n_b} f_a^{n_a} f_b^{n_b} f_c^{n_t - n_a - n_b}$.

Therefore, the probability of a cell of type A being in a specific droplet is given by

$q^A_{n_a, n_b, n_c} := P(\text{cell of type A, in droplet with } N_a = n_a, N_b = n_b, N_c = n_t - n_a - n_b)$,

$= \frac{1}{f_a \lambda} n_a \frac{\lambda^{n_t} e^{-\lambda}}{n_t!} \binom{n_t}{n_a, n_b, n_t - n_a - n_b} f_a^{n_a} f_b^{n_b} f_c^{n_t - n_a - n_b}$,

and similarly for cell-types B and C.

In the droplets, the cells will start to grow. Their eventual number of offspring depends on the initial fractions of the different cell-types. We denote the number of offspring A made per initial cell of A by G^A, which is thus dependent on the initial cell-type fractions: $G^A = G^A(n_a, n_b, n_c)$. Furthermore, this is also dependent of the final total number of cells in the droplet, which we call the carrying capacity: CC. If we have the formulae for G^A, G^B, G^C, we could calculate the average growth for each cell-type by

$$\langle G^A \rangle = \sum_{n_t=0}^{\infty} \sum_{n_a=0}^{n_t} \sum_{n_b=0}^{n_t-n_a} q_{n_a,n_b,n_c}^{A} \, G^A(n_a, n_b, n_t - n_a - n_b),$$

$$= \frac{1}{f_a \lambda} \sum_{n_t=0}^{\infty} \frac{\lambda^{n_t} e^{-\lambda}}{n_t!} \sum_{n_a=0}^{n_t} \sum_{n_b=0}^{n_t-n_a} n_a \binom{n_t}{n_a, n_b, n_t-n_a-n_b} f_a^{n_a} f_b^{n_b} f_c^{n_t-n_a-n_b}$$

$$\cdot \, G^A(n_a, n_b, n_t - n_a - n_b),$$

and similarly for cell-types B and C.

To determine G^A we make a few simplifying assumptions:

1. We assume that a certain part of the carrying capacity is facilitated by pairs of cooperators A, B. How large this carrying capacity is, is determined by the initial fractions: $CC_{\text{comm}} = CC_0 \frac{2\min\{n_a, n_b\}}{2\min\{n_a, n_b\} + n_c}$. This means that, the more cheaters there are, the less efficient the available substrate is used, and less offspring can be made.
2. The different cell-types are differentially dependent of this facilitated carrying capacity. With a cell-type-specific CC_{ind}^{A}, we can model that cells might even grow (although less) without pairs of cooperators around.
3. The cheater has a certain growth advantage, because it doesn't need to produce the costly public goods. We model this by assuming that the final cheater fraction is given by

   $$f_C^{\text{end}} = (1 + s_{\text{c}}) \frac{n_c}{n_a + n_b + n_c} - (s_c)\left(\frac{n_c}{n_a + n_b + n_c}\right)^2,$$

   where s_c is a parameter that quantifies the growth advantage of the cheaters. The maximal increase in cheater fraction is ¼ s_c, which is reached when $\frac{n_c}{n_a + n_b + n_c}$ equals 0.5.
4. The two cooperators are stoichiometrically coupled: they always end up in a 1:1-ratio. We therefore have $f_A^{\text{end}} = f_B^{\text{end}} = 1 - f_C^{\text{end}}/2$

Based on these assumptions, and with the aim of keeping the model as simple as possible, we end up with:

$$G^A(n_a, n_b, n_c) = \frac{1}{n_a}\left(CC_{\text{ind}}^{A}\frac{n_c}{2\min\{n_a,n_b\}+n_c} + CC_0\frac{2\min\{n_a,n_b\}}{2\min\{n_a,n_b\}+n_c}\left(1 - f_C^{\text{end}}/2\right)\right),$$

$$G^B(n_a, n_b, n_c) = \frac{1}{n_b}\left(CC_{\text{ind}}^{B}\frac{n_c}{2\min\{n_a,n_b\}+n_c} + CC_0\frac{2\min\{n_a,n_b\}}{2\min\{n_a,n_b\}+n_c}\left(1 - f_C^{\text{end}}/2\right)\right),$$

$$G^C(n_a, n_b, n_c) = \frac{1}{n_c}\left(CC_{\text{ind}}^{C}\frac{n_c}{2\min\{n_a,n_b\}+n_c} + CC_0\frac{2\min\{n_a,n_b\}}{2\min\{n_a,n_b\}+n_c}f_C^{\text{end}}\right).$$

We furthermore enforce that the above growth factors are at least one, because we do not include cell death in our models. Inserting these equations in the aforementioned expression for the average growth factor, we can find the growth of all cell-types as a function of the parameters.

New cell-type fractions can be calculated by multiplying the initial cell-type fractions by the average growth: $f_a\langle G_A\rangle$ and then normalizing the abundancies such that they add up to one.

Figure S3. Probabilistic model overview.

Step 1: Probability of droplets with 0, 1, …, n cells

Input

λ parameter $= \frac{\text{total cell number}}{\text{total droplet number}}$

Output

cells per droplet	probability (Poisson distribution, λ)
0	…
1	…
2	…
…	…
n	…

Step 2. Probability per cell-type combination

Input

Initial cell-type fractions

Probability of cells per droplet

Output

cells per droplet	cell combination	probability of specified combination
0	*not applicable*	*not applicable*
1	A<br>…<br>Z	P[A]<br>P[…]<br>P[Z]
2	AA<br>AB, BA<br>…<br>ZZ	P[AA]<br>P[AB], P[BA]<br>…<br>P[ZZ]
…	…	…
n	…<br>Z · n	…<br>P[Z · n]

probability per combination

Step 3. Simulate growth

Input

Droplets without cooperator pairs: independent growth capacity for each cell-type

Droplets with many cooperator pairs: 750 cells per droplet, cheaters have a 40% higher growth rate than cooperators

Output

cell combination	growth factor per combination
A<br>…<br>Z	f(A | A)<br>…<br>f(Z | Z)
AA<br>AB, BA<br>…<br>ZZ	f(A | AA)<br>f(A | AB), f(B | AB)<br>…<br>f(Z | ZZ)
…	…
…<br>Z · n	…<br>f(Z | ZZ)

Step 4. Average growth factor per cell-type

Input

Distribution of droplets with 0, 1, …, n cells.

Probability of combination

Growth factor per droplet

Output

cell-type	probability to be in combination, given the cell-type	growth factor per cell-type per combination	
A	P(A | A)<br>P(AA | A)<br>P(AB | A)<br>…<br>P(ZZ | A)	P(A | A) · f(A | A)<br>P(AA | A) · f(A | AA)<br>P(AB | A) · f(A | AB)<br>…<br>P(ZZ | A) · f(A | ZZ)	Average growth factor for A
…	…	…	Average growth factor for …
Z	P(A | Z)<br>P(AA | Z)<br>P(AB | Z)<br>…<br>P(ZZ | Z)	P(A | Z) · f(Z | A)<br>P(AA | Z) · f(Z | AA)<br>P(AB | Z) · f(Z | AB)<br>…<br>P(ZZ | Z) · f(Z | ZZ)	Average growth factor for Z

Step 5. Cell-type fractions after growth

Input

Initial cell-type fractions

Average growth factor per cell-type

Output

Cell-type abundancies=
initial cell-type fraction · average growth factor

New cell-type fractions = normalize cell-type abundancies such that they add up to 1.

Repeat from step 2

Section 5: Prediction of the optimal λ-value to enrich cooperators when cheaters are cooperation-dependent

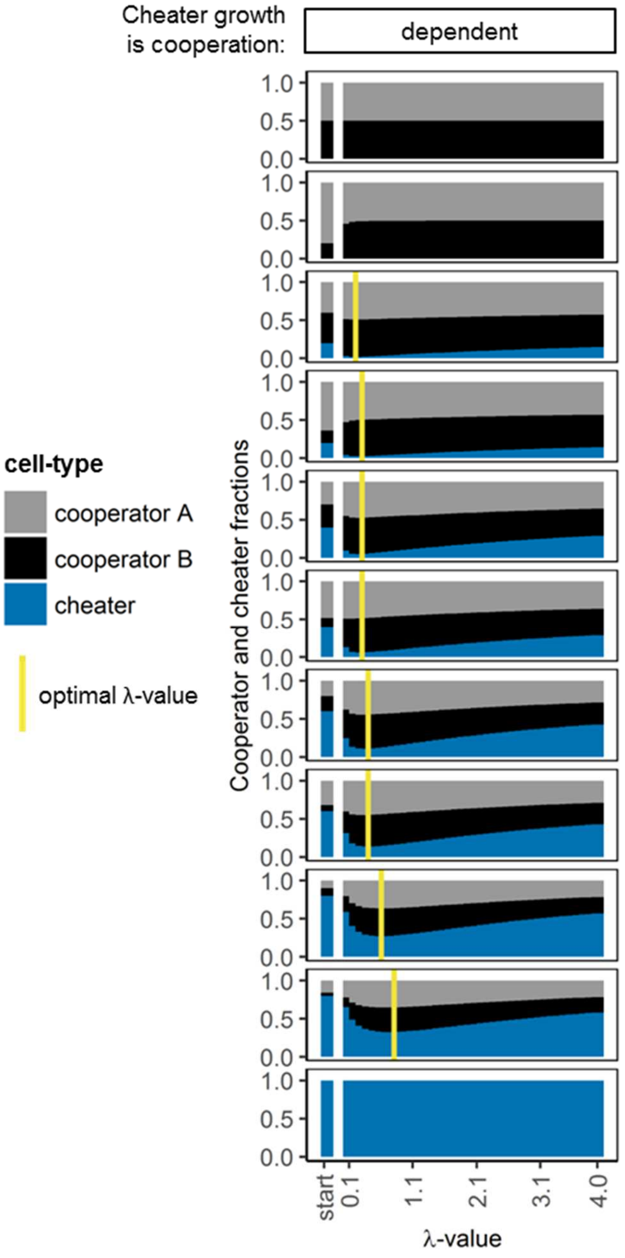

Figure S4. Predictions of the optimal λ-value when cheaters need public goods produced by cooperators to grow. Cooperator and cheater fractions after one propagation (y-axis) at a given value for the λ-parameter (x-axis). Each row corresponds to a different set of initial cooperator and cheater fractions, indicated by “start” on the x-axis. Yellow lines indicate at which λ-value the cheater fitness is minimized.

Section 6: Propagation in suspension

A synthetic consortium (Figure 2A) was propagated in a well-mixed suspension. The initial cooperator and cheater fractions were set the same as in Figure 2B. Figure S5 shows that after three consecutive propagations cheaters are enriched in the population (n=2).

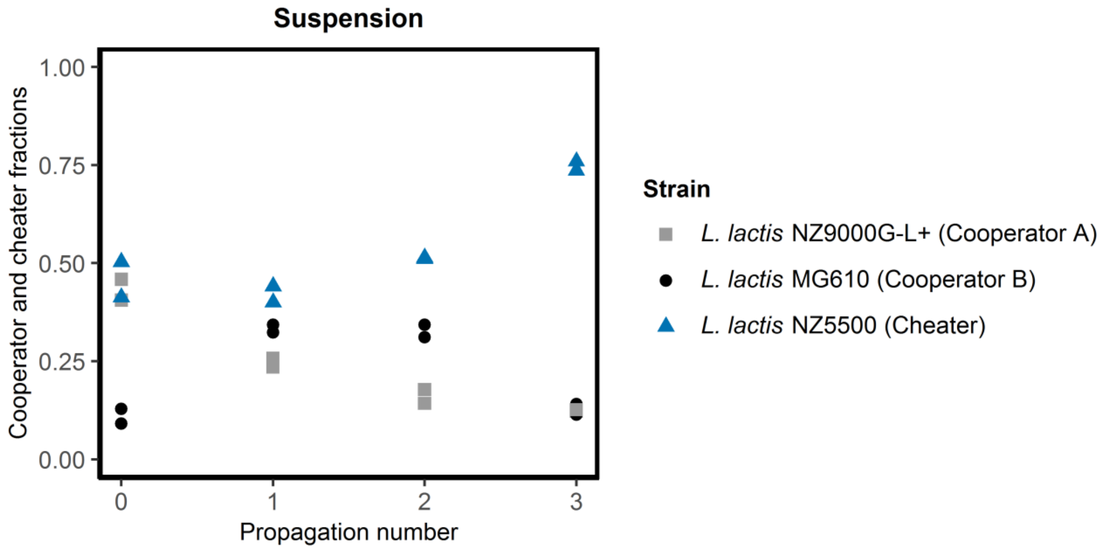

Figure S5. Propagation of cooperators and cheaters without spatial structure.

We used this data to estimate the growth advantage of cheaters in the presence of cooperators, parameter s_c in the model. In the model we assume that the final cheater fraction is given by: $f_C^{\text{end}} = (1 + s_c)\frac{n_c}{n_a+n_b+n_c} - (s_c)(\frac{n_c}{n_a+n_b+n_c})^2$ (supplementary information section 4). Figure S5 gives six combinations of initial and final cheater fractions, which allows us to fit s_c (Figure S6). We excluded n_b (*L. lactis* MG610) from the equation, as the cheater does not require glucose to grow, and its advantage is therefore mainly affected by the competition for amino acids with *L. lactis* NZ9000 Glc^-Lac^+. The obtained value for s_c is 0.52 ± 0.09.

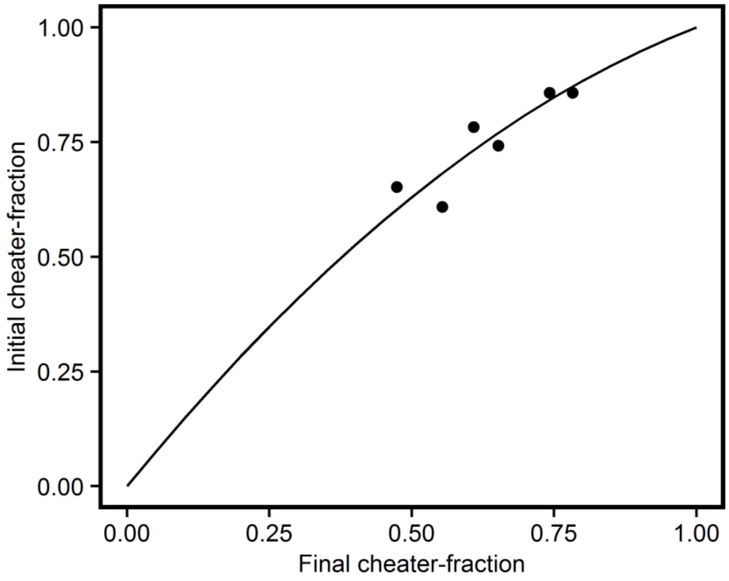

Figure S6. Estimation of the growth advantage of the cheater. Points indicate the measured cheater fractions during propagation in suspension (Figure S5). The solid line indicates the model-fit when s_c is set to 0.52.
